# Stretch force guides finger-like pattern of bone formation in suture

**DOI:** 10.1371/journal.pone.0177159

**Published:** 2017-05-04

**Authors:** Bo-Hai Wu, Xiao-Xing Kou, Ci Zhang, Yi-Mei Zhang, Zhen Cui, Xue-Dong Wang, Yan Liu, Da-Wei Liu, Yan-Heng Zhou

**Affiliations:** 1 Department of Orthodontics, Peking University School and Hospital of Stomatology, Beijing, P.R. China; 2 Center for Craniofacial Stem Cell Research and Regeneration, Peking University School and Hospital of Stomatology, Beijing, P.R. China; Medical University of South Carolina, UNITED STATES

## Abstract

Mechanical tension is widely applied on the suture to modulate the growth of craniofacial bones. Deeply understanding the features of bone formation in expanding sutures could help us to improve the outcomes of clinical treatment and avoid some side effects. Although there are reports that have uncovered some biological characteristics, the regular pattern of sutural bone formation in response to expansion forces is still unknown. Our study was to investigate the shape, arrangement and orientation of new bone formation in expanding sutures and explore related clinical implications. The premaxillary sutures of rat, which histologically resembles the sutures of human beings, became wider progressively under stretch force. Micro-CT detected new bones at day 3. Morphologically, these bones were forming in a finger-like pattern, projecting from the maxillae into the expanded sutures. There were about 4 finger-like bones appearing on the selected micro-CT sections at day 3 and this number increased to about 18 at day 7. The average length of these projections increased from 0.14 mm at day 3 to 0.81 mm at day 7. The volume of these bony protuberances increased to the highest level of 0.12 mm^3^ at day 7. HE staining demonstrated that these finger-like bones had thick bases connecting with the maxillae and thin fronts stretching into the expanded suture. Nasal sections had a higher frequency of finger-like bones occuring than the oral sections at day 3 and day 5. Masson-stained sections showed stretched fibers embedding into maxillary margins. Osteocalcin-positive osteoblasts changed their shapes from cuboidal to spindle and covered the surfaces of finger-like bones continuously. Alizarin red S and calcein deposited in the inner and outer layers of finger-like bones respectively, which showed that longer and larger bones formed on the nasal side of expanded sutures compared with the oral side. Interestingly, these finger-like bones were almost paralleling with the direction of stretch force. Inclined force led to inclined finger-like bones formation and deflection of bilateral maxillae. Additionally, heavily compressive force caused fracture of finger-like bones in the sutures. These data together proposed the special finger-like pattern of bone formation in sutures guided by stretch force, providing important implications for maxillary expansion.

## Introduction

Sutures are soft connective tissue, existing only between craniofacial bones of the skull. Mainly having two important functions, sutures serve as sites of active bone growth and provide the firm bond of union to allow a little movement for bones in response to mechanical force [1, 2]. Most sutures will experience various degrees of fusion by osseous union with aging [3].

Application of mechanical tension on the sutures before its closure can modulate the growth of craniofacial bones [4]. Maxillary expansion and protraction are the most dramatic examples of using tensile force for suture remodeling. They were characterized by accumulation of a large magnitude of stretch force in a short time to open the non-fused sutures and obtain new bone formation. However, there are some problems encountered in clinical treatments, such as pain, limited expansion outcomes and post-expansion relapse [5].

For the safety and effectiveness of clinical therapy, there have been many studies concerning the adaption of sutures upon stretch force. Studies performed in monkeys [6–8], pigs [9], cats [10], rabbits [11, 12] and murine animals [13–19] have reported similar biological responses to sutural tension, such as increased sutural width, angiogenesis, cell proliferation and new bone deposition. However, widespread observations of force-mediated suture remodeling, though of vital importance to our current understanding of suture adaption, fall short of addressing a fundamental question: what is the regular pattern of new bone forming in the suture? By understanding the pattern—the shape, alignment and orientation—of new bone formation in the suture under stretch force, it may help us to avoid some side effects and improve the outcomes of clinical treatment.

Actually, some studies have attempted to observe the features of bone formation using the midpalatal sutures of mice and rats as models [13, 15, 16]. However, it's worth pointing out that the midpalatal suture of murine animals, different from the suture of human beings, contain a large amount of cartilage. Admittedly, almost all sutures in human beings (after 3 years old) are exclusively fibrous interfaces mainly composed of fibroblasts, fibers, and vessels [20, 21].

In our study, we performed stretch force on the premaxillary, but not midpalatal, sutures of rats, which histologically resemble the sutures of human beings. We characterized the special finger-like pattern of bone formation in suture guided by tensile stimuli, focusing on the features of shape, arrangement and orientation of new bones. Further, we explored the significances of this bone formation pattern for maxillary expansion.

## Materials and methods

### Animals

5-week-old male Sprague-Dawley rats were used in our study. For histological and micro-CT analysis, 40 rats were randomly divided into 4 groups by different expansion periods: 1, 3, 5 or 7 days with 10 animals in each group, including 5 with expansion and 5 serving as sham-operations. For fluorescence bone labeling studies, an additional 6 animals were used with 3 for expansion and 3 for control. All animals were fed with powder food. All rats were euthanized in a CO2 gas chamber. Our study followed standards defined by the Care and Use of Laboratory Animals of China. All procedures were approved by the Institutional Animal Care and Use Committee of Peking University (No. LA 2013–92).

### Premaxillary expansion

We performed premaxillary expansion on rats adapted from previously reports [17]. Briefly, we fabricated helix springs with 0.014-inch Australian wire (TP Orthodontic Appliance Co. Ltd, WuXi, China). The force/deformation (F/Δ) ratio of the appliance has been calibrated using 3 springs activated at four force levels (A in S1 Fig). The force was measured using a force gauge, and the amount of deflection was determined using a ruler. After anesthetizing the rats with an intraperitoneal injection of 1% pentobarbital, we used a dental drill to make two grooves on the mesial sides of the maxillary incisors and then fitted a helix spring in the grooves (for applying inclined force, the grooves were made not on the same level). Light cured composite resin (3M Unitek, Monrovia, CA, USA) was added to fix the spring (B in S1 Fig). The initial expansion force was 1.76 ± 0.05N. The control rats were sham operated with broken springs without force. According to the anatomy of incisors and surrounding maxillary bones of rats, the rotation center was inferior to the suture. So, the incisor-maxillae complex moved in a slight tipping manner while the whole suture was expanded. We expanded the rats for 7 days and the sutures were not expanded excessively. For applying compressive force, the springs were cut and the maxillae were pressed heavily toward each other. Their weights were monitored daily. The inter-incisor distance was measured at hour 3, 6, 9 and 12 and day 1, 3, 5 and 7.

### Micro-computed tomographic evaluation

Animals were euthanized. Their maxillae were harvested and fixed in 10% neutral-buffered formalin for 24 hours and scanned coronally using high-resolution Micro-CT (Inveon MMCT, Siemens, USA). Raw image data was used to generate three-dimensional reconstructions and two-dimensional coronal sections. The suture width was calculated by measuring the distance between the frontal margins of two palatal bones. The number, length and volume of newly formed bones in the suture were calculated.

### Fluorescence labeling and sample processing

Rats were subcutaneously administered alizarin red S (30 mg/kg, Sigma) and calcein green (5 mg/kg, Sinopharm) on days 0 and 6 after expansion respectively, and sacrificed on day 7. The samples were fixed in 10% neutral-buffered formalin, and then embedded in polymethylmethacrylate (PMMA) without decalcification. Finally, specimens were cut into sections with a Leica SP1600 saw microtome. Sequential fluorescence labeling of the sections was observed using a Zeiss laser scanning microscope (LSM 510). Related measurements were carried out by software NIS-Elements D 4.00.03.

### Histology and histochemistry

After micro-CT scanning, the samples were demineralized in 15% (w/v) ethylenediaminetetra-acetic acid (EDTA) for 6 weeks, and then embedded with paraffin. Finally, they were cut into 5 μm thick sections and stained with HE and Masson. The angles of inclination for incisor and maxillary bones were measured and calculated. The frequency of finger-like bones per mm at day 3, day 5 and day 7 was calculated. According to the manufacturer’s protocol, serial sections from each group were stained for TRAP using a leukocyte acid phosphatase kit (387A, Sigma). These outcomes were observed using an inverted microscope system (Eclipse 80i, Nikon, Tokyo, Japan), and microphotographs were taken and measured using software NIS-Elements D 4.00.03.

### Immunochemistry staining

5-μm-thick sections were dewaxed in xylenes and rehydrated in ethanol baths. Sections were enzyme-treated for antigen retrieval with Trypson and 20 μg/ ml Proteinase K at 37°C for 30 min and then blocked with 5% fetal calf serum at room temperature for 30 min. Sections were incubated with anti-osteocalcin (Abcam Inc., MA, USA) primary antibodies at 4°C overnight. Positive immunoreactivity was detected using a 2-step DAB detection kit (Zhongshan Golden Bridge Biotechnology, Beijing, China) according to the manufacturer’s instructions. Controls for each antibody consisted of incubation with secondary antibody in the absence of primary antibody. Sections were counterstained with hematoxylin for 4 min followed by 10 min in running water. Photomicrographs were taken by an inverted microscope system (Eclipse 80i, Nikon, Tokyo, Japan). The length of osteoblasts and maxillary margins was measured by software NIS-Elements D 4.00.03.

### Statistical analysis

Statistical analyses were performed using the Statistic Package for Social Study (SPSS) version 20 software package (SPSS Inc., Chicago, Illinois, USA). To compare the differences between the control and expansion groups at each time point, unpaired Student’s t-test was used when the data was Gaussian distribution; otherwise Mann-Whitney Rank sum test was used. To compare the differences over time in each group, the one-way analysis of variance was performed followed by the least significant difference multiple comparison test. The significance level of the statistical analysis was set at *p* < 0.05 or *p* < 0.01.

## Results

### Finger-like pattern of new bone formation in expanded sutures

The rats tolerated the expansion springs well. No significant difference was observed in body weights between the two groups throughout the experimental period (C in S1 Fig). The inter-incisor width of expanded rats increased quickly to about 2 mm within the first day; and at later time points, the rate of increase decreased and remained nearly constant (D in S1 Fig).

**Fig 1:**
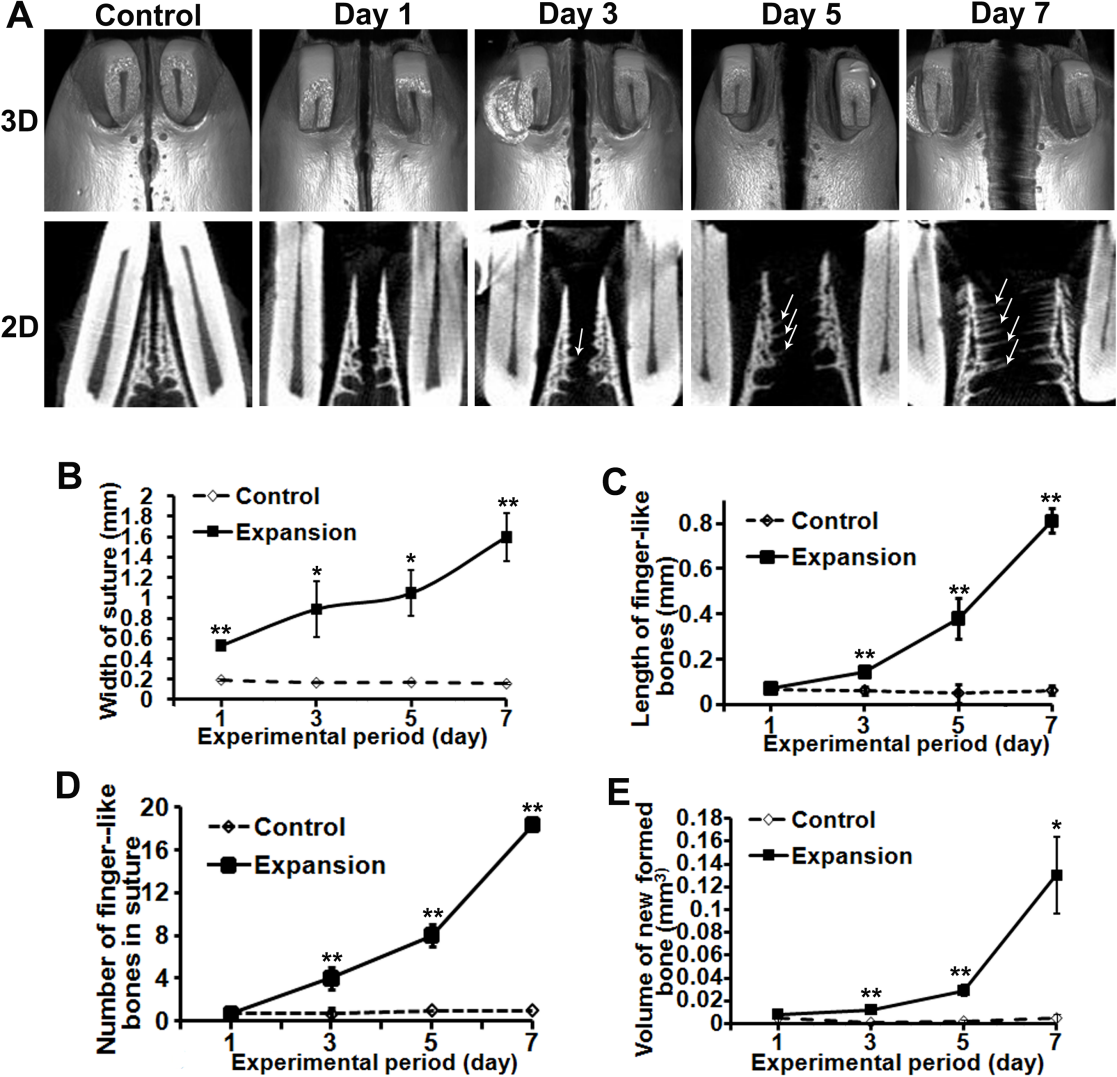
Micro-CT detected new bones forming in finger-like pattern in the expanded sutures. (A) Occlusal view of three-dimensional (3D) reconstructed maxillae at different expansion stages (upper panel). The two-dimensional (2D) coronal sections, oriented with oral side up and nasal side down, are displayed in the lower panel. Arrows point to the new bone forming in finger-like pattern. (B) The width of sutures increased from day 1 to day 7 under expansion force. (C) The length of these finger-like bones increased following the expansion period. (D) The number of finger-like bones increased from day 3. (E) The total volume of newly formed finger-like bones in the sutures did not changed at day 1, but increased from day 3 to day 7 during the expansion period. *Statistically significant difference between the control and expansion groups (*p < 0.05, **p < 0.01).

We scanned the maxillae by micro-CT to observe the sutural width change and new bone formation under stretch force. In the control group, the sutural width did not significantly change over time. However, in the expansion group, the sutural width increased from day 1 to day 7 (Fig 1A and 1B). The sutural margins of control rats were not flat but had small bony convexes. They did not appear to experience adaption over time. For the expansion rats, no visible new bone formation was observed in the sutures at day 1. However, small protuberances were obvious at day 3. The average length of these projections increased from 0.14 mm at day 3 to 0.81 mm at day 7 (Fig 1A and 1C). There was a space between two adjacent protuberances. Morphologically, we called these bony protuberances “finger-like bones” (Fig 1A, solid arrows). There were about 4 finger-like bones appearing on the selected micro-CT sections at day 3 and this number increased to about 18 at day 7 (Fig 1A and 1D). Compared with the control rats, the volume of bony protuberances in the sutures did not change at day 1, but increased to 0.012 mm^3^ at day 3 (Fig 1E). The volume dramatically increased to the highest level of 0.12 mm^3^ at day 7.

**Fig 2:**
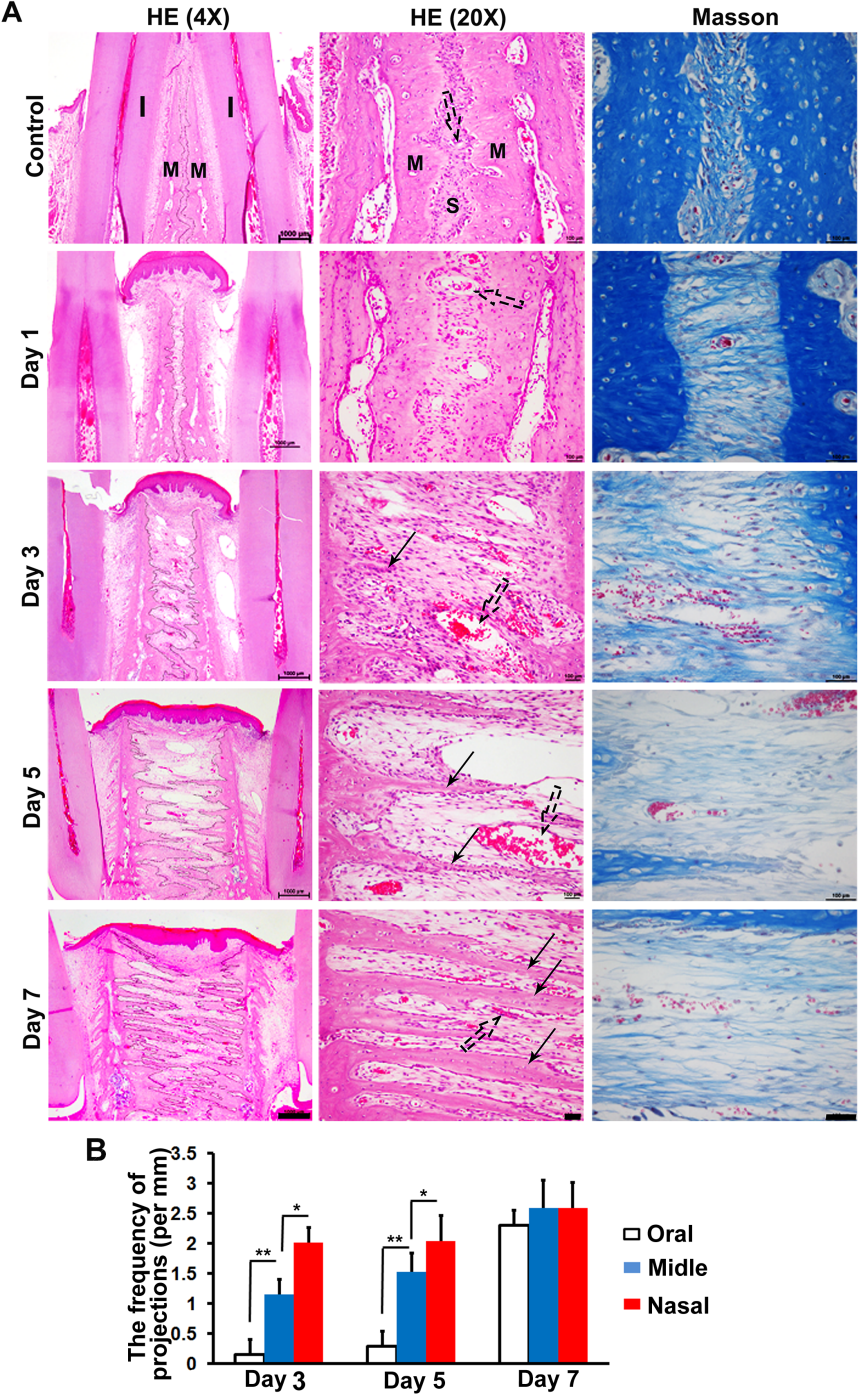
Histology of finger-like bones in the expanded suture. (A) Left panels of HE staining show that each of the new bones was almost triangle-shaped with a thick base connecting with the maxillae and thin front stretching into the expanded sutures (solid arrows). There was a space between two adjacent newly formed bones. The finger-like bones from one side of the maxillae did not contact or fuse with the finger-like bones stretching from the other side. The right panel of Masson staining shows that the ends of fibers were embedding into the margins of maxillary bones. They became progressively straighter and less dense, aligning in the direction of the stretch force. (B) The frequency of finger-like bones on different sections of expanding sutures at day 3, day 5 and day 7. All images are oriented with oral side up and nasal side down. The sutural margins in the left panel of (A) were traced using black lines. I = Incisor, M = Maxillae, S = Suture. *p < 0.05, **p < 0.01. Scale bars from left to right are 1000 μm, 100 μm and 100 μm separately.

Next, we observed histological sections. Similar to the micro-CT outcomes, we observed small bony convexes on the maxillary margins in control suture (Fig 2A). Interestingly, the concave between two convexes often contained one blood vessel (Fig 2A, dotted arrows). The sutures in the control animals did not undergo significant change during the experimental period. However, both the incisors and maxillae in the expansion rats were inclined outside under the expansion force (S2 Fig). No new bone formation was observed in the opened suture at day 1. Obviously, some triangle-shaped protuberances, with wide bases connecting with the maxillae and pointed fronts projecting into the sutures, were seen at day 3 (Fig 2A, solid arrows). These finger-like bones became longer and longer from day 3 to day 7. Interestingly, the fingers from one side of the maxillary bone did not contact or fuse with those from the other side during the experimental period. The frequency of finger-like bones projecting from the maxillary margin varied on different sections of suture at day 3 and day 5 (Fig 2B). Generally, there were more finger-like projections per mm occurring on the nasal section of sutures compared with the oral section. However, all the oral, middle and nasal sections have the similar frequency of new bony projections at day 7 (Fig 2B).

Masson staining showed that in the sutures of control rats, the fibers were curly and connecting bilateral maxillae (Fig 2A, right panel). However, these fibers started to be straight and aligned parallel to the direction of the distraction force from day 1 in the expansion group. Also, these elongated fibers gradually became sparse following the expansion period. No obvious broken fibers were observed during expansion.

### Spindle-shaped osteoblasts covered the surface of finger-like bones continuously

**Fig 3:**
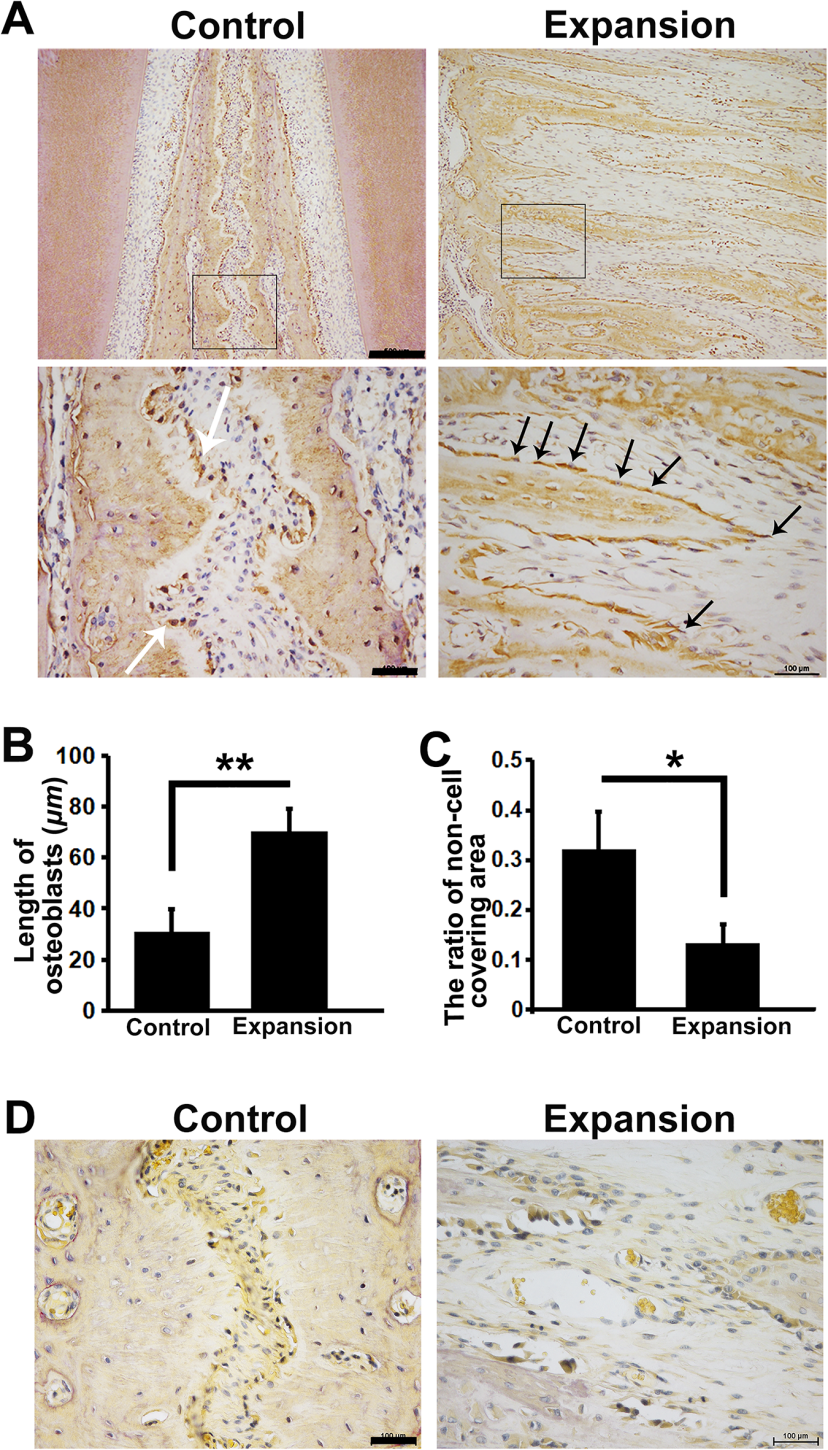
Spindle-shaped osteoblasts continuously covered finger-like bones. (A, B, C) In the control sutures, osteoblasts with OCN expression (white arrows) had cuboidal shapes and were discontinuously distributed on the margin of maxillae. In the expanded sutures, these OCN positive cells (black arrows) changed to be spindle-shaped and were continuously covering the surfaces of newly formed finger-like bones. These OCN positive cells oriented in the direction of the expansion force. Boxed areas in the upper panel are shown magnified in the lower panel respectively. The ratio of non-cell covering = the length of non-cell covering margins/the length of total margins. (D) TRAP staining shows no visible osteoclasts occurring in either the control or the expanded sutures. Scale bars from up to down are 500 μm, 100 μm and 100 μm separately. *p < 0.05, **p < 0.01.

To observe the distribution of osteoblasts in the suture, we stained sections with anti-osteocalcin (OCN) antibody. Interestingly, in the control sutures, osteoblasts with OCN expression were cuboidal and discontinuously distributed along the margin of the maxillary bone. In the expanded suture, a layer of OCN positive osteoblasts were in spindle shapes and continuously covering the surfaces of newly formed finger-like bones (Fig 3A, 3B and 3C). These osteoblasts oriented in the direction of the expansion force. In order to determine if osteoclasts also participated in this suture remodeling process, we did TRAP staining. However, no visible osteoclasts were found in control and expanded sutures from day 1 to day 7 (Fig 3D).

### Different sizes of finger-like bones forming in different segments of expanded sutures

**Fig 4:**
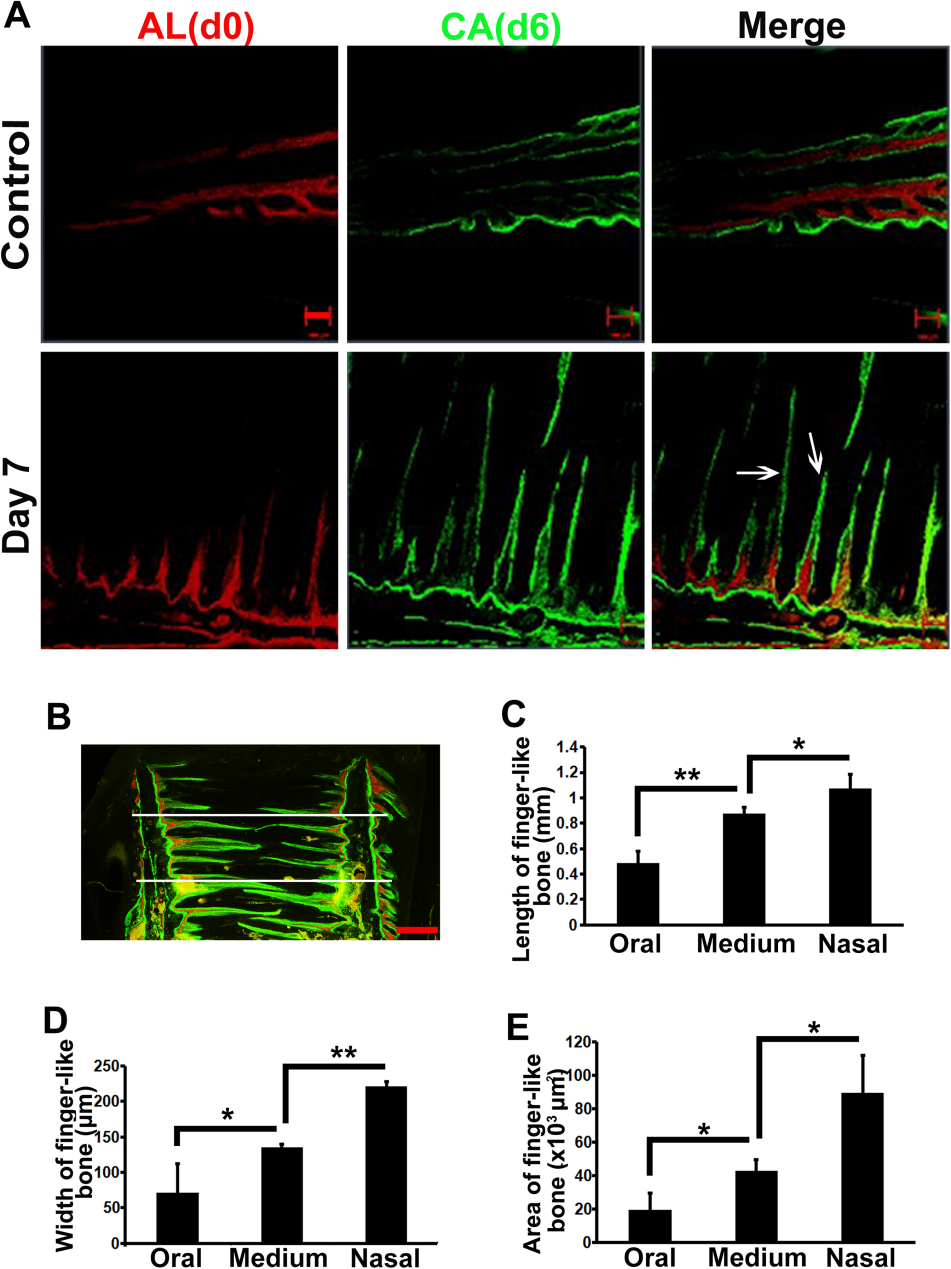
Different sizes of finger-like bones forming in different segments of expanded sutures. (A) Coronal sections of sequential fluorescence labeled premaxillary sutures are oriented with oral side left and nasal side right. Alizarin red S (red) administered at day 0 (d0) labeled the inner core of newly formed bones. Calcein (green) injected at day 6 (d6) labeled the outer layers of newly deposited bones. Arrows point to the newly formed finger-like bones, projecting from the maxillae into the expanded suture. (B, C) The length of finger-like bones increased from the oral side to medium segment to nasal side. (B, D) The width of the base of new bones increased from oral side to medium segment to nasal side. (B, E) The area of finger-like bones was also augmented. Scale bars are 100 μm in (A) and 500 μm in (B). *p < 0.05, **p < 0.01.

To dynamically observe the new bone formation in the sutures in vivo, rats were administered separately with alizarin red S and calcein green at the beginning of expansion and one day before sacrifice. Compared with the control rats, active finger-like bone formation and mineralization occurred in the widened sutures, as evidenced by fluorescence deposition (Fig 4A). Alizarin red S was deposited in the inner core of newly formed finger-like bones. Calcein appeared on the outer layer of the new bones. Interestingly, different segments of the expanded sutures have different characteristics of finger-like bone formation (Fig 4B). The length, width and area of finger-like bones increased from the oral section to medium segment to nasal side (Fig 4B, 4C, 4D and 4E). This indicates that a larger volume of new bone was forming on the nasal side than on the oral side of sutures.

### The direction of force determined the orientation of finger-like bone and maxillae

**Fig 5:**
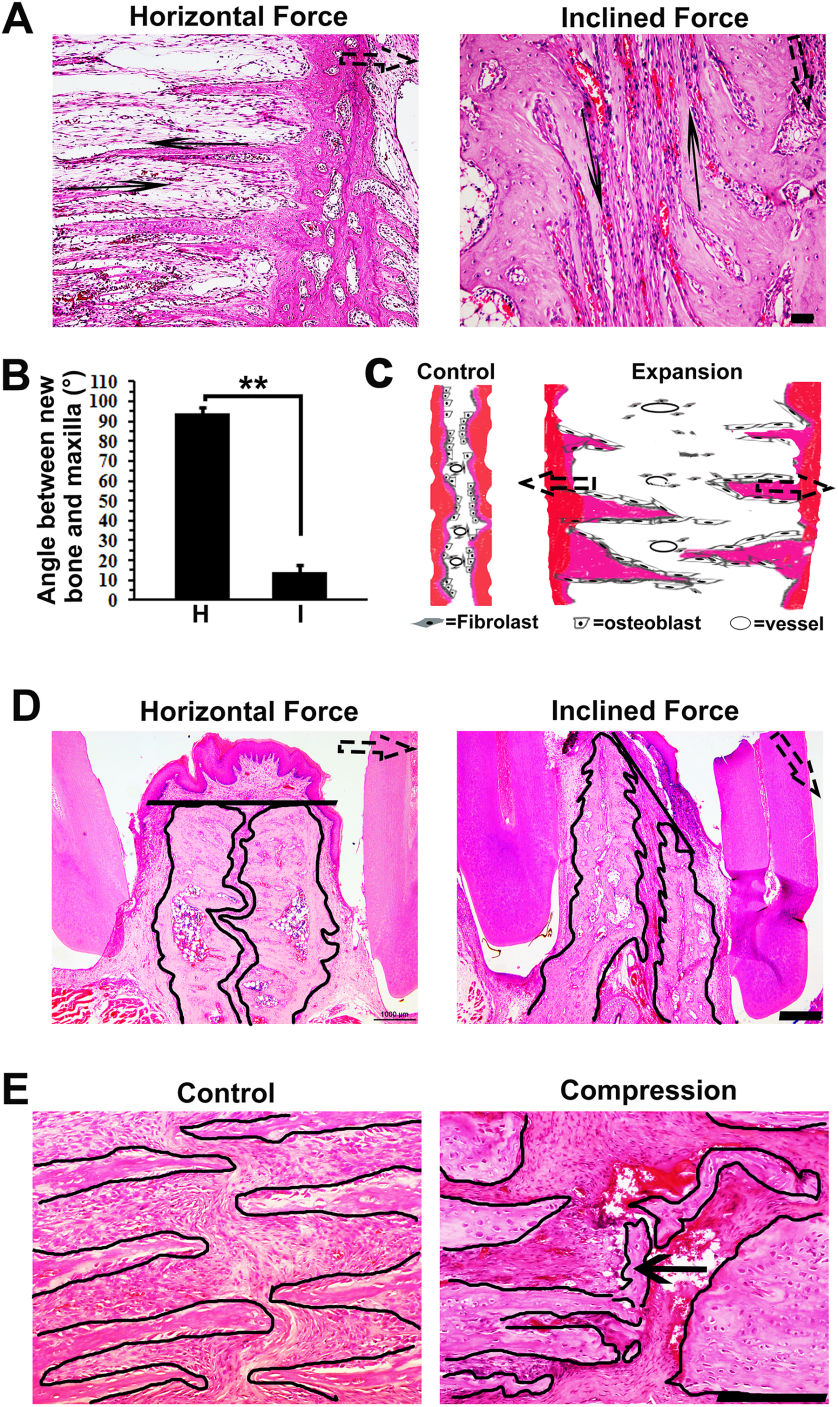
The direction of force determined the orientation of finger-like bone and maxillae. (A) The finger-like bones were horizontal and parallel to the horizontal stretch force (left). After inclined force was applied, the finger-likes bones changed their direction (solid arrows) to follow the direction of inclined stretch force (right). The dotted arrow represents the direction of stretch force. (B) In the inclined force group, the angle between finger-like bones and maxillae decreased. (C) Diagrams elucidate the sutural remodeling pattern under tensional force. The premaxilary sutures of natural growing rats (left) were rugged and had some small bony prominences. Cuboidal-shaped osteoblasts were discontinuously distributed on the margins of maxillary bones. After performing stretch force (right), new bones were forming in finger-like patterns and parallel with the direction of stretch force (dotted arrows). A layer of spindle-shaped osteoblasts continuously covered the surface of these finger-like bones, accompanied by vessels expansion. (D) The inclined force caused deflection of two blocks of maxillae. The maxillary margins are outlined by dark lines. The oral fronts of bilateral maxillary bone are connected by a straight dark line. The dotted arrow represents the direction of stretch force. (D) Compressive force led to fracture of finger-like bones in the suture (arrow). The finger-like bones are outlined by dark lines. The dotted arrow represents the direction of stretch force. Scale bars are 100 μm in (A), 1000 μm in (D) and 200 μm in (D). *p < 0.05, **p < 0.01.

We observed that the finger-like bones were almost parallel with each other. In order to test if the orientation of the finger-like bones was determined by the direction of the expansion force, we inclined the stretch force applying on the incisors. Interestingly, the finger-like bones were also inclined to follow the direction of expansion force (Fig 5A). The angles between the finger-like bones and the maxillae decreased in the inclined expansion group (Fig 5B). All the data presented above totally suggest an orientational finger-like pattern of bone formation in expanded sutures, which is accompanied by transformation of osteoblasts (Fig 5C).

Further, we observed the deflection of bilateral maxillae under inclined stretch force (Fig 5D). The two maxillae located front and back after expansion. Interestingly, the direction of deflection almost matched the direction of the stretch force. Additionally, constriction was often applied on the suture during clinical expansion. In order to explore possible effects of constrictive force on the finger-like bones in sutures, we applied heavy compressive force on bilateral maxillae after expansion. Obviously, fracture occurred on the front of finger-like bones in the expanded sutures (Fig 5E).

## Discussion

In our current study, we demonstrated the finger-like pattern of bone remodeling in premaxillary sutures of rats under mechanical tension. A large amount of finger-like bones, continuously covered by spindle-like osteoblasts, were projecting from bilateral maxillary bones to the direction of the tensional strain. This process was accompanied by stretched fibers. To our knowledge, this is the first proposal about the orientational finger-like pattern of bone formation in sutures under mechanical tension. Furthermore, we found that inclined expansion force could guide inclined finger-like bones formation in sutures, leading to deflection of two blocks of maxillae. Additionally, constrictive force could cause fracture of these finger-like bones during expansion. These findings may provide potential implications for our clinical practice.

For decades, sutures have been known as important growth sites for cranial bones. Many studies have applied different kinds of tensile forces on the suture to explore their effects on bone formation. Liu and colleagues [22] found that continuous forces were more effective than intermittent forces to induce bone deposition in expanding sutures. For the magnitude of tensile force, they found larger forces could produce greater amounts of bone formation [23]. Interestingly, it seems that the cyclic forces could stimulate sutural bone deposition more effectively than static forces [24]. In our study, we applied continuous and relatively large amount of force and observed obvious bone formation and finger-like patterns. In further studies, we may test different kinds and forms of expansion forces to explore their influences on the pattern of bone formation in sutures.

After placement of springs, we noted a series of changes consistent with the reactions observed in clinical sutural expansion treatment [25]. We reason that at the start of expansion, the force acted on the incisors directly. As a result, the incisors separated from each other quickly because that the soft periodontal tissue surrounded them was easy to change its size. Next, the tensile force was passed from periodontal tissue to maxillary bones over time. Gradually, the sutural fibers stretched and elongated in the direction of tensional force. As a result, the bilateral maxillary bones began to tilt laterally with the suture becoming wider [18].

In the expanding sutures, the new bones were forming in “finger-like” patterns. Notably, the sutures of human beings are not straight but tortuous [26, 27]. In our study, the margins of unexpanded premaxillary sutures of young rats were also concavo-convex with small bony protrusions, which is analogous to the histology of human beings’ sutures [20, 21]. Researchers have thought that the wavy shape of suture may be caused by tensile force and shear strain from the intracranial pressure and masticatory muscle mass [28–30]. Matching with this speculation, we observed the interlocking of finger-like prominences quickly and obviously after applying a relatively large magnitude of force on the suture. Further study is needed to explore how the expansion force leads to this finger-like pattern. We suppose that this is caused by non-uniform expansion forces distribution in the suture. As described above, the stretch force passed from the fibers embedded in the maxillary margins induced deposition of new bone on their fronts. In our experiment, we noted that vessels were almost located in the concaves of suture. Maybe, these vessels interrupt the force delivering by fibers.

Besides, we noted that the nasal section of sutures has a higher frequency of finger-like bones occurring than the oral sections at day 3 and day 5. At day 7, there were longer and bigger finger-like bones formed on the nasal side of expanded sutures compared with the oral side. This phenomenon may help to explain that the sutures have different timing of closure [31–34], which is referred to as the “zipper” mechanism of sutural closure where fusing occurred first on one side of the suture [35]. Why there were more new finger-like bones forming on the nasal section of suture than the oral section is open to question. We speculate that it may be caused by different magnitude of force distribution in the expanding suture. We noted that the expansion force was separating the suture in a slight tipping manner and the oral section of sutures was expanded wider than the nasal section. It seems to indicate that there was a larger force delivering on the oral section than the nasal section. However, there has been a study showing that larger force could produce greater amount of bone formation [23]. So it seems that this inequality of bone formation in expanding sutures could not be attributed to the different magnitude of force distribution. Further study is needed to explore if the nature of different sections of suture determines the amount of finger-like bones formation.

Interestingly, we noted that after applying stretch force, the osteoblasts in the suture changed their cuboidal shapes into spindle-like and continuously covered the surfaces of newly forming finger-like bones. Many studies have revealed that stretch force could stimulate osteoblasts and induce bone formation [36–38]. We speculate that the tensional force in the fibers of sutures was transmitted to the osteoblasts on the fronts of maxillae, and then the osteoblasts transformed into active condition and began to produce matrix and form new bones [17, 39, 40]. In our study, no osteoclast was observed in the suture during expansion. This indicates that the suture was not experiencing resorption at this early stage.

This finger-like pattern of bone formation in expanding sutures may provide some implications for our current clinical practice. Even though suture expansion could effectively extend the width of maxillae, relapses often occur [5]. The inequality of bone formation in expanding sutures observed in our study may partly explain this irritating clinical problem. For example, the new bone in the nasal section matured quickly after rapid expansion, however, new bone in the oral section was forming later. So, the retention of suture should be long enough so that the sections with delayed bone formation could make up the lag in new bone deposition after expansion. Moreover, some researchers have concerns that suture expansion may facilitate maxillary displacement [41]. Indeed, we found that inclined stretch force could cause inclined finger-like bone formation in the sutures, which led to the deflection of bilateral maxillary halves. It may indicate that we should apply horizontal expansion force on patients, avoiding deviated maxillary bone adaption. Also, compressive force was often applied on patients during expansion to produce greater disarticulation [42]. We observed that the new forming finger-like bones were thin and long, so it was not a surprise to note that these finger-like bones experienced fracture under heavy compressive force in our study. This finding reminds us to pay attention to the magnitude of force and apply moderate compressive force during suture expansion, especially in the early stages of expansion.

## Supporting information

S1 Fig The model of premaxillary suture expansion in rats. (A) The force-deflection (F/Δ) curve of springs delivering open force. After recording the force of 3 springs at 4 deformation levels, linear regression analysis was performed to create the trend line (R^2^ = 0.9911). The initial deformation of springs in our experiments was about 9 mm, so the initial expansion force was about 1.76 N. (B) Diagram shows that the spring was bonded on the middle point of maxillary incisors’ crown. The potential rotation center is pointed out. (C) There was no statistically difference in body weights between the expansion and sham-operated groups during the experimental period. (D) The incisors space increased quickly within the first expansion day. However, the rate of increase slowed and remained almost constant during the following experimental period. *Statistically significant difference between the control and compression groups (*p < 0.05, **p < 0.01).
(TIF)

S2 Fig The inclination of maxillae and incisors during expansion. (A) The angles between incisors and horizontal line increased at all stages of expansion. (B) The angles between alveolar bones and horizontal line increased in all time points of expanded sutures compared with the control group. *Statistically significant difference between the control and compression groups (*p < 0.05, **p < 0.01).
(TIF)
