# Supplementary figures and images for: Stretch force guides finger-like pattern of bone formation in suture
Source: PLoS One. 2017 May 4;12(5):e0177159. doi: 10.1371/journal.pone.0177159 (PMC5417680; doi:10.1371/journal.pone.0177159)

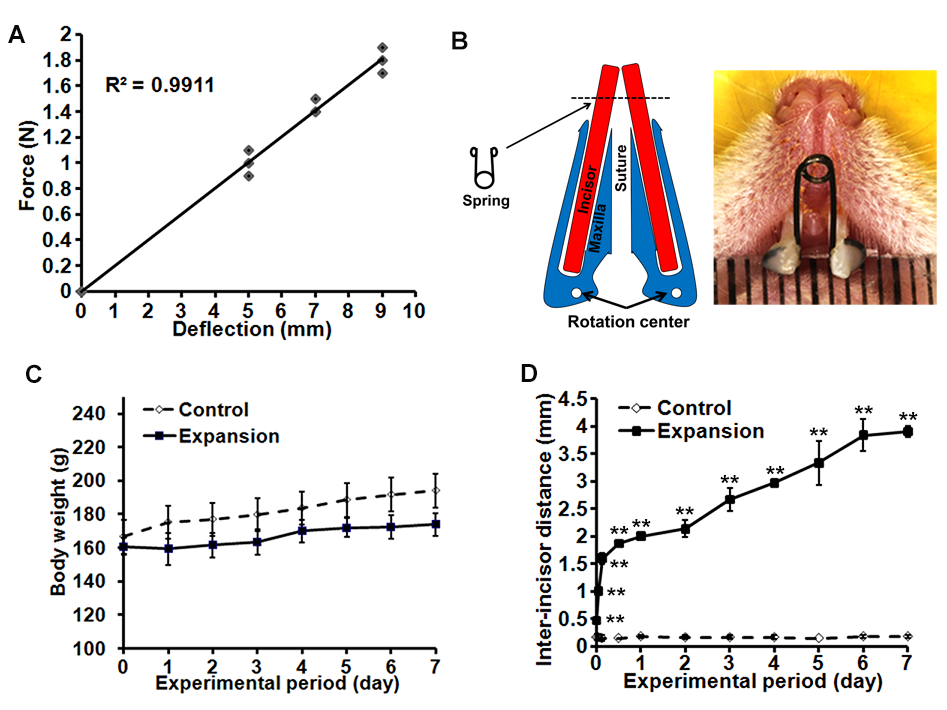

Supplement: S1 Fig — (A) The force-deflection (F/Δ) curve of springs delivering open force. After recording the force of 3 springs at 4 deformation levels, linear regression analysis was performed to create the trend line (R2 = 0.9911). The initial deformation of springs in our experiments was about 9 mm, so the initial expansion force was about 1.76 N. (B) Diagram shows that the spring was bonded on the middle point of maxillary incisors’ crown. The potential rotation center is pointed out. (C) There was no statistically difference in body weights between the expansion and sham-operated groups during the experimental period. (D) The incisors space increased quickly within the first expansion day. However, the rate of increase slowed and remained almost constant during the following experimental period. *Statistically significant difference between the control and compression groups (*p < 0.05, **p < 0.01). (TIF) [file pone.0177159.s001.tif]

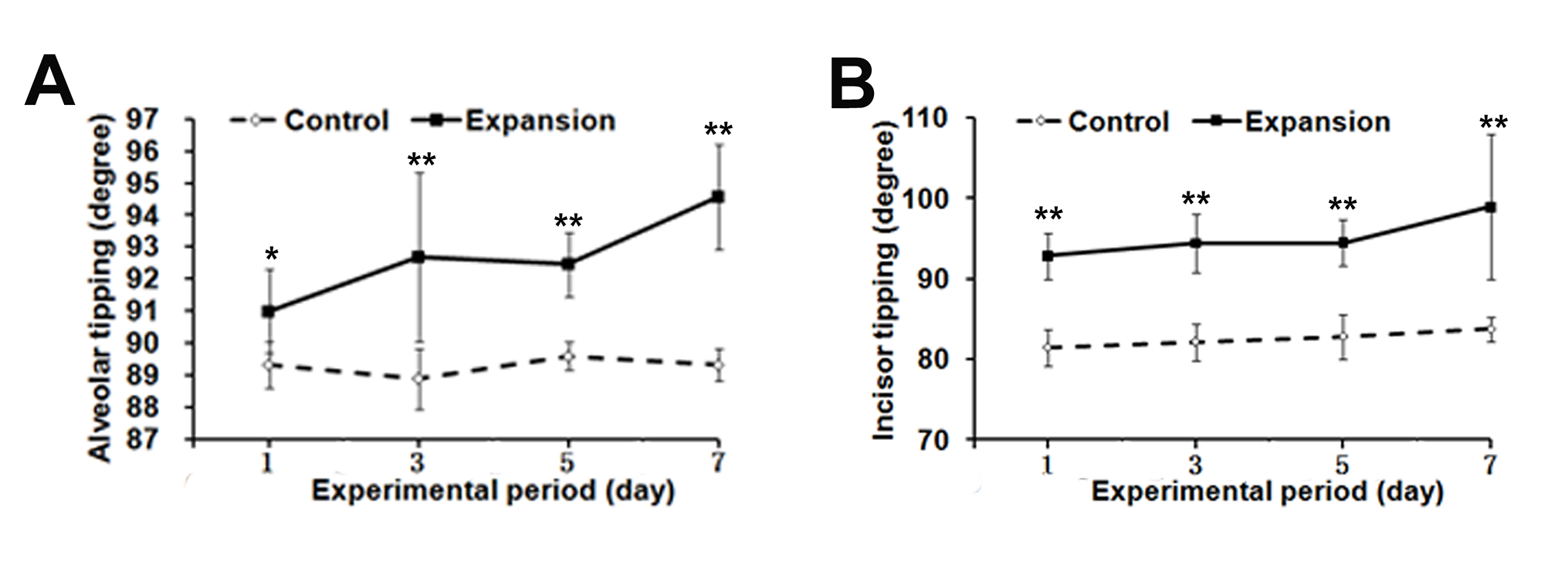

Supplement: S2 Fig — (A) The angles between incisors and horizontal line increased at all stages of expansion. (B) The angles between alveolar bones and horizontal line increased in all time points of expanded sutures compared with the control group. *Statistically significant difference between the control and compression groups (*p < 0.05, **p < 0.01). (TIF) [file pone.0177159.s002.tif]
